# Impact of COPD on clinical and CT characteristics of COVID-19-associated pneumonia: single tertiary center experience

**DOI:** 10.1186/s43055-022-00932-8

**Published:** 2022-11-29

**Authors:** Yevgeniya Filippenko, Marianna Zagurovskaya, Aigul Abdrakhmanova, Saule Kassenova, Zhanar Zhakenova, Aizat Aimakhanova, Zhamilya Zholdybay

**Affiliations:** 1 Department of Diagnostic Radiology, Kazakh National Medical University Named After S.D. Asfendiyarov, Tole bi St. 94, 050000 Almaty, Kazakhstan; 2 Department of Radiology and Imaging Sciences, Indiana University, Indianapolis, USA; 3 Zhekenova City Clinical Hospital of Infectious Diseases, Almaty, Kazakhstan; 4 Department of Internal Medicine, Scientific Research Institute of Cardiology and Internal Diseases, Almaty, Kazakhstan; 5 Department of Biostatistics and Basis of Scientific Analysis, Kazakh National Medical University Named After S.D. Asfendiyarov, Almaty, Kazakhstan

**Keywords:** COVID-19, Pneumonia, Chronic obstructive pulmonary disease, Computed tomography

## Abstract

**Background:**

The severe acute respiratory syndrome-related coronavirus 2 pandemic continues to this day worldwide. Individuals with COPD are at increased risk of contracting SARS-CoV-2. Most of the conducted studies are based on the clinical assessment of COVID-19 infection with different comorbidities. The specific contribution of COPD to the severity of the disease and outcome still remains the point of investigation. The main goals of our study are to assess COPD’s influence on the severity of clinical and CT characteristics of COVID-19 pneumonia and associated in-hospital mortality.

**Results:**

This is a retrospective study on 281 patients with RT-PCR-confirmed COVID-19 infection and CT spectrum of COVID-19 pneumonia. Fifty patients have COPD based on CT criteria. No significant difference was observed in the mean hospital length of stay, arterial oxygen saturation on admission or in-hospital mortality between COPD and non-COPD groups. Patients with COPD were two times less likely to have fever less than 37.9** °C** (RR = 2.037; 95% CI 1.114–3.724, *p* = 0.016), but higher absolute neutrophil count (*p* = 0.033) and median level of neutrophil/lymphocyte ratio (*p* = 0.029). The COPD group was presented with milder CT severity score (especially CT1, less than 25% of lung involvement) (*p* = 0.022), less likely to have bilateral (RR = 2.601; 95% CI: 1.341–5.044, *p* = 0.023) or central (RR = 1.754; 95% CI 1.024–3.003, *p* = 0.046) distribution of ground-glass opacities, right lower lobe (RR = 2.887; 95% CI 1.574–5.293, *p* = 0.008) or left lung (RR = 2.627; 95% CI 1.448–4.765, *p* = 0.009) involvement, and “crazy-paving” pattern (RR = 2.208; 95% CI 1.292–3.774, *p* = 0.003). Both moderate positive and negative relationship was observed between CT1, CT4, hypoxia and in-hospital mortality in the COPD group (*r* = − 0.383, *p* = 0.033; *r* = 0.486, *p* = 0.007; *r* = − 0,354, *p* = 0,022, respectively).

**Conclusion:**

The presence of COPD by imaging criteria in the settings of COVID-19-associated pneumonia did not significantly influence the clinical or imaging performance of the patients, nor was it linked to the increased in-hospital mortality.

## Background

The severe acute respiratory syndrome-related coronavirus 2 (SARS-CoV-2) pandemic continues to this day worldwide. Survival and clinical outcomes for the Coronavirus disease (COVID-19) infection are reported to be less favorable for the elderly and patients with comorbidities [1, 2]. The severity of the disease ranges from asymptomatic infections to severe pneumonia with respiratory failure or death [3].

Chronic obstructive pulmonary disease (COPD) is the third leading cause of death worldwide, the overall 5-year survival for COPD patients is between 56 and 92% depending on the severity of the disease [4, 5]. COPD includes airway inflammation and remodeling, with variable alveolar destruction (emphysema) [6]. COPD patients suffer from dyspnea, cough and sputum production, and may experience sudden worsening (exacerbations) that are often caused by respiratory tract infections [7]. Individuals with COPD are reported to have an increased risk of contracting SARS-CoV-2 and present with worse outcomes due to chronically impaired lung function and frequent coexistence with other comorbidities, such as hypertension, obesity and ischemic heart disease [8–10]. COPD prevalence in COVID-19 infection is reported to be in the range of 2% to 12% [10, 11]. No significant differences in the incidence of fever, cough or sputum production are reported between COPD and non-COPD patients [12].

Thoracic computed tomography (CT) and reverse transcription-polymerase chain reaction (RT-PCR) are the mainstay in the diagnosis of COVID-19-associated pneumonia regardless of the presence of COPD, with CT sensitivity for the disease outperforming that of RT-PCR, 94–98% vs. 47–83.3%, respectively [13–15]. Most of the conducted studies are based on the clinical assessment of the severity of COVID-19 infection with complex comorbidities [16–19]. The specific contribution of COPD to the severity of the disease and outcome still remains the point of investigation.

The objective of our study was to evaluate COPD influence on the severity of clinical, laboratory and CT characteristics of COVID-19-associated pneumonia on initial evaluation, as well as in-hospital mortality.

## Methods

### Study design and subjects

This retrospective study was approved by the Institutional Review Board and waived for informed consent. Consecutive nonprobability sampling was done. Between October 1 and December 2, 2020, we analyzed chest CT examinations of 798 in-patients with RT-PCR-confirmed COVID-19 infection from our tertiary Infectious Disease Hospital (Almaty, Kazakhstan). A total of 281 patients (145 men and 136 women) over the age of 40 years with the CT spectrum of COVID-19-associated pneumonia were included to the study. Fifty patients (men = 40, women = 10) with a history of smoking demonstrated CT findings compatible with COPD: emphysema (centrilobular, paraseptal, panlobular, bullous), features of respiratory bronchiolitis (centrilobular nodules, mosaic attenuation), abnormal bronchi (expansion of the lumen, bronchial wall thickening) [20]. Exclusion criteria were: age younger than 40 years, lack of lung changes indicative of COVID-19 infection, significant motion artifacts on CT images, or large pleural effusion leading to a significant lobe(s) atelectasis, lung cancer or history of lung surgery.

Spirometry and pulmonary function testing were avoided due to an active infection and increased risk of SARS-CoV-2 droplet transmission [21, 22]. Symptoms of respiratory failure were determined by the value of arterial oxygen saturation (SpO2) ≤ 95% and the presence of dyspnea at rest. The management for both COPD and non-COPD groups was similar and based on the oxygen parameters and clinical performance. Available demographic, clinical and laboratory data were extracted from de-identified electronic medical records. Demographic data included sex, age and years of smoking, duration of hospital stay and in-hospital mortality. Clinical data included symptoms upon evaluation, SpO2 and history of comorbidities. White blood cells (WBC) and absolute lymphocyte counts (ALC), absolute neutrophil count (ANC), neutrophil/lymphocyte ratio (NLR), levels of alanine aminotransferase (ALT) and aspartate aminotransferase (AST), total protein, prothrombin time and fibrinogen concentration were considered for the laboratory analysis.

### Imaging techniques

All CT chest examinations were performed using a CT machine with 64 channels (CT Revolution EVO, GE Healthcare, USA). Acquisition CT parameters included tube voltage of 120 kVp; tube current of 60–120 mAs with automatic exposure control; slice thickness of 1.25 mm; and reconstruction interval of 1.0–3.0 mm. CT images were obtained with the patient in the supine position at full inspiration without the use of an intravenous contrast medium.

### Image analyses

The CT images were evaluated with both lung (width, 1500 HU; level, − 600 HU) and mediastinal (width, 400 HU; level, 40 HU) window settings by two radiologists with 6 and 35 years of experience in thoracic CT imaging. Typical (ground-glass opacities (GGO), bilateral alveolar lower lobe peripheral involvement, vascular enlargement) and less specific (central lung involvement, consolidation, crazy-paving pattern, fibrous stripe, reversed halo sign, pleural effusion, mediastinal lymphadenopathy) chest CT findings previously described with COVID-19 pneumonia were included into the correlative analysis [23].

The severity of the disease by the CT assessment and extension of COVID-19-related lung involvement was visually scored, according to the previously published an “empirical” visual scale from 0 to 5 [24, 25]. Thus, each lobe has received the following score: 1 for 1%–4%, 2 for 5%–25%, 3 for 26%–49%, 4 for 50%–75%, and 5 for 76%–100% of the alveolar involvement. CT severity score (maximum 100%) was calculated as the sum of individual lobe scores (maximum 25 points) multiplied by 4. CT-derived severity of COVID-19 infection was based on the total lung scores from 1 to 4 as follows: CT 1 (mild) < 25%; CT 2 (moderate) 25–50%; CT 3 (medium-to-severe) 50–75%; CT 4 (severe) > 75% of the total lung involvement.

### Statistical analysis

Statistical analyses were performed using SPSS Statistics version 28 (IBM Corp., Armonk, NY, USA). Quantitative data that obeyed a normal distribution were presented in the form M ± σ, where M is the average value and σ is the standard deviation. The data were quantified and compared between the two groups using the Student’s t test for unpaired samples, Pearson's chi-squared test, Fisher's exact test, odds ratios (ORs) and 95% confidence intervals (CIs). Kendall rank correlation coefficient method was applied to study the association between hypoxia, in-hospital mortality and CT severity score (CT 1–4). Interpretation of results was performed in accordance with the Chaddock scale. P values < 0.05 were considered to indicate a statistically significant difference.

## Results

### Demographics, smoking history and comorbidities

A total of 281 patients from two groups, COPD (n = 50) and non-COPD (n = 231), were included in the analysis. Details on demographics and health status characteristics of the study population are summarized in Table 1. There was a significant men prevalence in the COPD group, 80.0% (n = 40) vs. 20% (n = 10) of women in comparison with the non-COPD group, where women represented 54.5% (n = 126) vs. 45.4% (n = 105) of men (*p* < 0.001). Regardless the gender, COPD patients were slightly older, 68.4 ± 9.8 years vs. 63.86 ± 11.1 years in the non-COPD group (*p* = 0.008). All COPD patients had a tobacco smoking history contrary to the non-COPD group, where only 45 smokers (19.5%) were identified. Patients with COPD had a long history of smoking, 34.22 ± 12.85 years vs. 27.57 ± 10.49 years of the non-COPD group (*p* = 0.007). The rates of arterial hypertension, ischemic heart disease, diabetes mellitus, or stroke did not reveal significant differences between the groups.

**Table 1:** Demographic and relevant health status characteristics (n = 281)

Data	COPD (*n* = 50)	Non-COPD (*n* = 231)	OR (95% CI)	*p* value
**Demographic information**				
Men	40 (80.0%)	105 (45.5%)	0.208 (0.099–0.437)	< 0.001*
Women	10 (20.0%)	126 (54.5%)		
Age, years (mean age)	44–85 (68.4 ± 9.8)	41–93 (63.86 ± 11.1)		0.008*
Tobacco smoking, years	34.22 ± 12.85	27.57 ± 10.49**		0.007*
Hospital length of stay, days	10.02 ± 6.92	11.64 ± 6.34		0.108
In-hospital mortality	7 (14.0%)	21 (9.1%)	0.614 (0.246–1.536)	0.300
**Relevant clinical symptoms**				
Fever ≥ 38.0 °C	26 (52.0%)	98 (42.4%)	0.680 (0.368–1.256)	0.216
Fever 37.0–37.9 °C	12 (24.0%)	98 (42.4%)	2.333 (1.159–4.696)	0.016*
No fever (< 37.0 °C)	12 (24.0%)	35 (15.2%)	0.565 (0.269–1.188)	0.128
Dyspnea at rest	34 (68.0%)	125 (54.1%)	0.555 (0.290–1.061)	0.072
Dry cough	29 (58.0%)	156 (67.5%)	1.506 (0.806–2.815)	0.198
Arterial oxygen saturation (SpO_2)_, %, on admission	91.84 ± 5.40	91.85 ± 6.58		0.986
**Comorbidities**				
Arterial hypertension	34 (68.0%)	145 (62.8%)	0.793 (0.414–1.522)	0.486
Ischemic heart disease	18 (36.0%)	92 (39.8%)	1.177 (0.624–2.220)	0.615
Diabetes mellitus	6 (12.0%)	49 (21.2%)	1.974 (0.795–4.902)	0.137
Stroke history	6 (12.0%)	18 (7.8%)	0.620 (0.233–1.650)	0.400

Data are presented as median (IQR) or n (%). CI—confidence interval; OR—odds ratio*;* COPD—chronic obstructive pulmonary disease

* Statistically significant *p* < 0.05. ** Out of 281 patients only 45 with smoking experience

### Duration of hospitalization and in-hospital mortality

Hospital stay duration did not differ significantly between the groups, 10.02 ± 6.92 vs. 11.64 ± 6.34 days in the COPD vs. non-COPD group, respectively (p = 0.108). In-hospital mortality showed a higher tendency in COPD patients, 14.0% vs. 9.1% in non-COPD patients, without, however, statistical significance (OR = 0.614; 95% CI 0.246–1.536, *p* = 0.300).

### Symptoms and oxygen saturation

The spectrum of clinical symptoms between the two groups did not differ statistically, except for the fever of less than 37.9 °C. Thus, the patients from the COPD group were two times less likely to have such a degree of fever than the non-COPD group (RR = 2.037; 95% CI 1.114–3.724, *p* = 0.016). No significant differences in the SpO2 on admission (*p* = 0.986) were observed between COPD (91.84 ± 5.4%) and non-COPD groups (91.85 ± 6.6%).

### Laboratory values analysis

Hematological and biochemical laboratory parameters of the groups are included in Table 2. No significant difference in WBC and ALC levels was observed between COPD vs. non-COPD group: WBC, 7.73 ± 4.94 × 109/L vs. 6.33 ± 3.6 × 109/L (*p* = 0.064); ALC, 1.19 ± 0.63 × 109/L vs. 1.33 ± 0.99 × 109/L (*p* = 0.343), respectively. However, the median level of ANC was higher in the COPD group, 6.06 ± 4.68 × 109/L vs. 4.54 ± 3.22 × 109/L in non- COPD group (*p* = 0.033). Moreover, a higher median level of NLR in the COPD group was observed, 6.46 ± 6.41 vs. 4.34 ± 3.98 in the non-COPD group (*p* = 0.029). The median level of ALT, AST, total protein, prothrombin time and fibrinogen concentration between the two groups did not differ significantly.

**Table 2:** Relevant laboratory values

Data	COPD (*n* = 50)	Non-COPD (*n* = 231)	*p* value
WBC, × 10^9^/L	7.73 ± 4.94	6.33 ± 3.6	0.064
ALC, × 10^9^/L	1.19 ± 0.63	1.33 ± 0.99	0.343
ANC, × 10^9^/L	6.06 ± 4.68	4.54 ± 3.22	0.033*
NLR	6.46 ± 6.41	4.34 ± 3.98	0.029*
ALT, U/L	36.13 ± 26.06	39.2 ± 36.74	0.575
AST, U/L	36.56 ± 22.27	39.67 ± 27.81	0.461
Total protein, g/L	66.99 ± 5.75	67.57 ± 7.33	0.597
Prothrombin time, s	14.71 ± 2.71	13.9 ± 4.57	0.253
Fibrinogen, g/L	4.61 ± 2.18	4.19 ± 1.79	0.208

ALC—absolute lymphocyte count; ALT—alanine aminotransferase; ANC—absolute neutrophil count; AST—aspartate aminotransferase; COPD—chronic obstructive pulmonary disease; NLR—neutrophil/lymphocyte ratio; WBC—white blood cells

* Statistically significant *p* < 0.05

### Imaging findings and associated severity scores

**Fig. 1:**
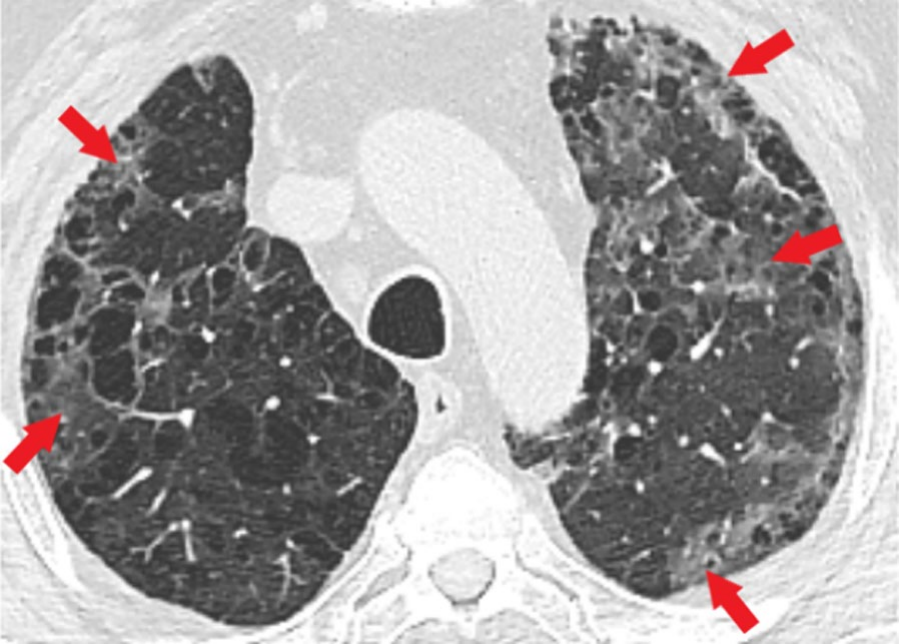
A 71-year-old man in the COPD group. Axial CT image at the level of upper lobes shows bilateral distribution of peripheral and subpleural predominant GGO (red arrows) in the background of paraseptal and centrilobular emphysema. COPD—chronic obstructive pulmonary disease*;* CT—computed tomography; GGO—ground-glass opacities

**Fig. 2:**
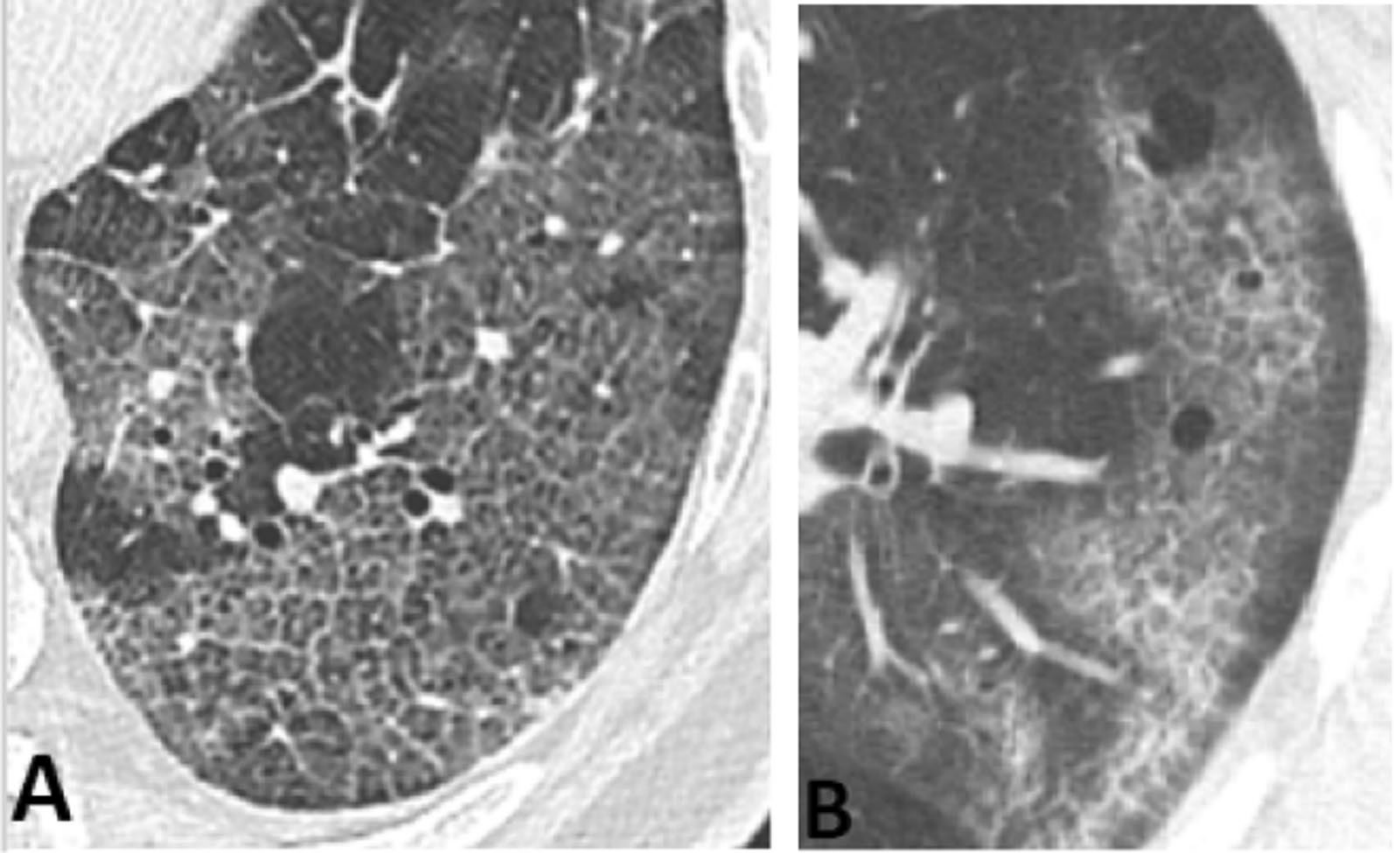
**A** a 70-year-old man without COPD/emphysema. **B** a 58-year-old man with COPD/emphysema. On axial CT images (region of interest) “crazy-paving” pattern. COPD—chronic obstructive pulmonary disease; CT—computed tomography

**Fig. 3:**
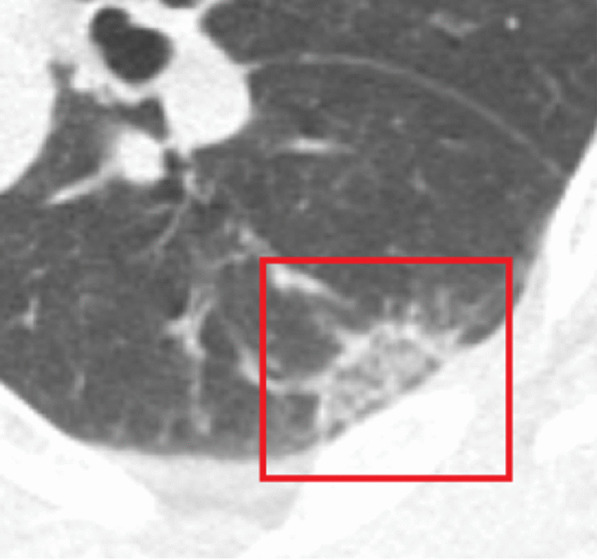
A 73-year-old man in the non-COPD group. Axial CT image (region of interest), demonstrates “reversed halo” sign (red frame). COPD—chronic obstructive pulmonary disease; CT—computed tomography

**Fig. 4:**
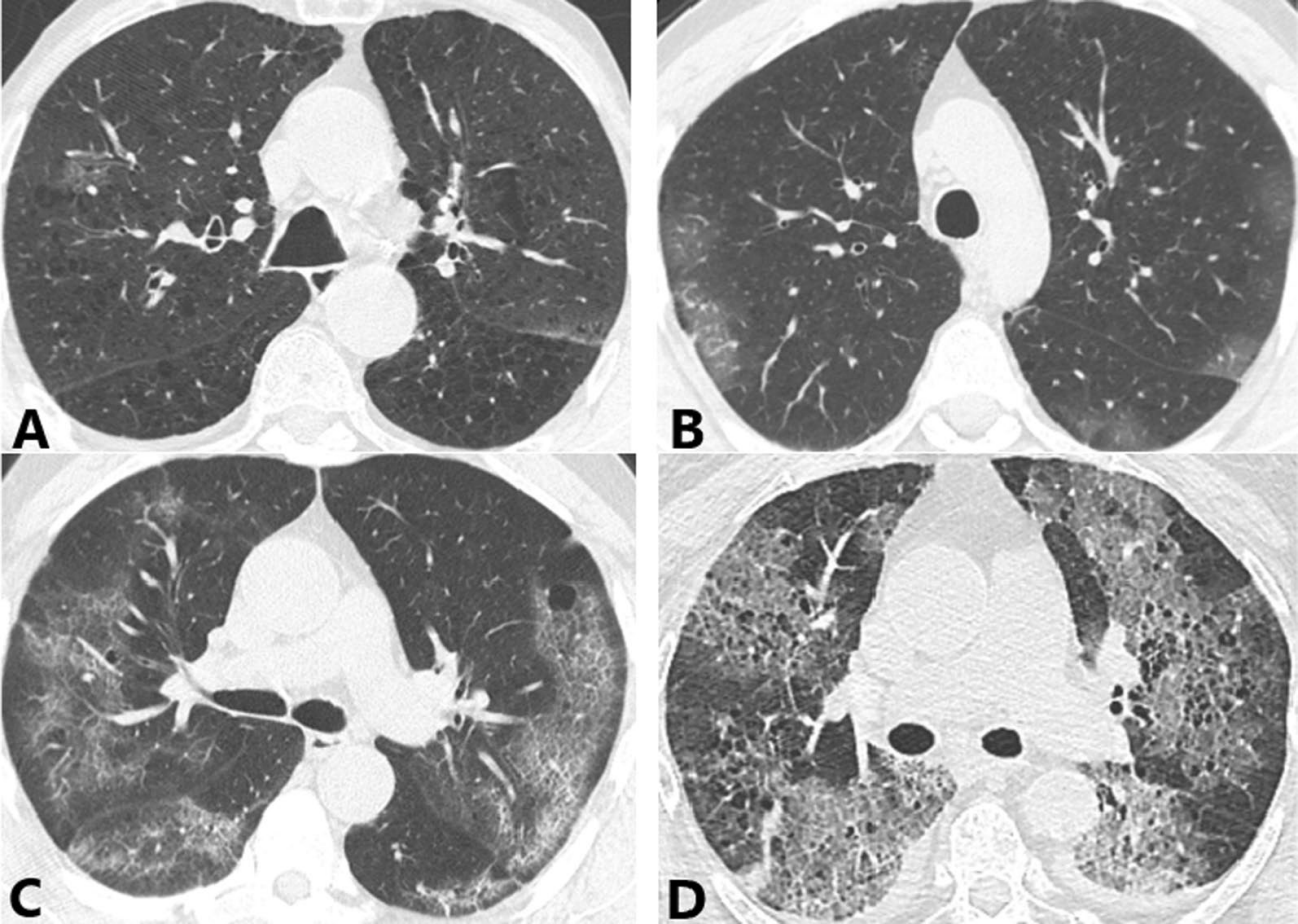
Illustration of total lung scores of patients in the COPD group shown on axial CT images. **A** CT 1 (mild), < 25% involvement. **B** CT 2 (moderate), 25–50% involvement. **C** CT 3 (medium-to-severe), 50–75% involvement. **D** CT 4 (severe), > 75% involvement. COPD—chronic obstructive pulmonary disease; CT—computed tomography

Table 3 demonstrates CT findings and distribution of abnormalities in both the groups. Thus, predominant bilateral distribution of GGO was observed in both groups, 88.0% in COPD and 96.5% in non-COPD group. Peripheral GGO was present in 96.0% of COPD vs. 99.1% non-COPD group (Fig. 1). Patients with COPD were 2.6 and 1.7 times less likely to have bilateral (RR = 2.601; 95% CI 1.341–5.044, *p* = 0.023) and central lung involvement. Unilateral distribution of GGO was more often observed in the COPD group (12.0% vs. 3.5%, *p* = 0.023). Moreover, patients with COPD by CT imaging criteria had 2.8 and 2.6 times less probability to have right lower lobe (RR = 2.887; 95% CI 1.574–5.293, *p* = 0.008) and left lower lobe (RR = 2.627; 95% CI 1.448–4.765, *p* = 0.009) involvement in comparison with the non-COPD group. Vascular enlargement was a very common finding regardless of the COPD status, 84.0% in COPD and 88.3% non-COPD group. "Crazy-paving" pattern and fibrous stripes were more common in non-COPD group, 57.1% and 35.5%, vs. 34.0% and 24.0% in COPD group, respectively. Thus, patients with COPD were 2.2 times less plausible to have a “crazy-paving” pattern (Fig. 2) (RR = 2.208; 95% CI 1.292–3.774, *p* = 0.003). “Reversed halo” sign (Fig. 3) was rare and presented only in the non-COPD group, 3.5%. Pleural effusion was observed 3 times more common in the COPD group, yet seen only in 10% of the patients. Patients in the non-COPD group were 1.3 times less apparent to have enlarged mediastinal lymph nodes (RR = 1.308; 95% CI 1.172–1.460, *p* < 0.001). According to the empirical visual scale and specific categories (Fig. 4), the severity of lung involvement in COPD vs. non-COPD group was as the following: CT 1, 38.0% vs. 22.5%; (*p* = 0.022); CT 2, 34.0% vs. 35.9%; CT 3, 20.0% vs. 28.1%; CT 4, 8.0% vs. 12.9%. Incidence of diffuse alveolar damage was very low in groups, 2% in COPD- and 2.6% in non-COPD groups.

**Table 3:** Relevant CT chest findings

Data	COPD (*n* = 50)	Non-COPD (*n* = 231)	OR (95% CI)	*p* value
**GGO distribution**				
Bilateral	44 (88.0%)	223 (96.5%)	3.801 (1.257–11.497)	0.023*
Unilateral	6 (12.0%)	8 (3.5%)	0.263 (0.087–0.796)	0.023*
Peripheral	48 (96.0%)	229 (99.1%)	4.771 (0.656–34.710)	0.147
Central	36 (72.0%)	194 (83.9%)	2.039 (1.002–4.149)	0.046*
Right upper lobe	43 (86.0%)	216 (93.5%)	2.344 (0.902–6.092)	0.084
Right middle lobe	40 (80.0%)	205 (88.7%)	1.971 (0.882–4.405)	0.093
Right lower lobe	43 (86.0%)	223 (96.5%)	4.538 (1.563–13.171)	0.008*
Left upper lobe	46 (92.0%)	219 (94.8%)	1.587 (0.490–5.141)	0.498
Left lower lobe	42 (84.0%)	220 (95.2%)	3.810 (1.446–10.036)	0.009*
**Other abnormalities**				
"Crazy-paving" pattern	17 (34.0%)	132 (57.1%)	2.615 (1.378–4.963)	0.003*
Fibrous stripes	11 (22.0%)	82 (35.5%)	1.951 (0.948–4.014)	0.066
"Reversed halo"	0	8 (3.5%)		0.358
Vascular enlargement	42 (84.0%)	204 (88.3%)	1.439 (0.611–3.387)	0.403
Pleural effusion	5 (10.0%)	7 (3.0%)	0.281 (0.085–0.926)	0.043*
Mediastinal lymphadenopathy	42 (84.0%)	107 (46.3%)	0.164 (0.074–0.365)	< 0.001*
Diffuse alveolar damage	1 (2.0%)	6 (2.6%)	1.307 (0.154–11.100)	1.000
**CT severity score**				
CT 1	19 (38.0%)	52 (22.5%)	0.474 (0.248–0.907)	0.022*
CT 2	17 (34.0%)	83 (35.9%)	1.089 (0.572–2.073)	0.796
CT 3	10 (20.0%)	65 (28.1%)	1.566 (0.740–3.316)	0.238
CT 4	4 (8.0%)	30 (12.9%)	1.716 (0.576–5.112)	0.327

CI—confidence interval; COPD—chronic obstructive pulmonary disease; CT—computed tomography; GGO—ground-glass opacities; OR—odds ratio

* Statistically significant *p* < 0.05

### Correlation of hypoxia, CT severity score and in-hospital mortality

Seven lethal outcomes due to respiratory failure and oxygen saturation below 95% on admission were seen in the COPD group. In the non-COPD group, the lethal outcome was observed in 21 cases, with decreased oxygen saturation in 19 patients. Table 4 summarizes the correlation between hypoxia, in-hospital mortality and CT severity score in COPD and non-COPD groups. Thus, 32 patients in the COPD- and 138 patients in the control group had hypoxia (SpO2 < 95%) on admission. Both moderate positive and negative relationship was observed between CT1 and CT4 scores, hypoxia, and in-hospital mortality in the COPD group (r = − 0.383, p = 0.033; r = 0.486, *p* = 0.007; r = − 0.354, *p* = 0.022, respectively). This correlation was also significant in non-COPD group between CT4 score, hypoxia, and in-hospital mortality (r = 0.261, p = 0.002; r = − 0.248, *p* = 0.001), as well as CT2/CT4 sores and hypoxia (r = 0.184, *p* = 0.012; r = − 0.288, *p* = 0.000).

**Table 4:** Correlative analysis of hypoxia, in-hospital mortality and CT-based severity score

	COPD (*n* = 32)		non-COPD (*n* = 138)	
Variables	In-hospital mortality	Hypoxia	In-hospital mortality	Hypoxia
CT 1	*n* = 0	*n* = 11	*n* = 1	*n* = 20
	*r* = − 0.383*	*r* = 0.204	*r* = − 0.105	*r* = 0.107
	*p* = 0.033*	*p* = 0.187	*p* = 0.220	*p* = 0.146
CT 2	*n* = 3	*n* = 10	*n* = 5	*n* = 45
	*r* = 0.133	*r* = − 0.025	*r* = − 0.054	*r* = 0.184*
	*p* = 0.461	*p* = 0.870	*p* = 0.530	*p* = 0.012*
CT 3	*n* = 1	*n* = 7	*n* = 6	*n* = 49
	*r* = − 0.097	*r* = − 0.099	*r* = − 0.077	*r* = − 0.030
	*p* = 0.589	*p* = 0.520	*p* = 0.369	*p* = 0.683
CT 4	*n* = 3	*n* = 4	*n* = 9	*n* = 24
	*r* = 0.486*	*r* = − 0.133	*r* = 0.261*	*r* = − 0.288*
	*p* = 0.007*	*p* = 0.389	*p* = 0.002*	*p* = 0.000*
Hypoxia	*n* = 7	*-*	*n* = 19	*-*
	*r* = − 0.354*		*r* = − 0.248*	
	*p* = 0.022*		*p* = 0.001*	

COPD—chronic obstructive pulmonary disease; CT—computed tomography; r—Kendall's Tau (Kendall Rank Correlation Coefficient)

* Statistically significant *p* < 0.05

## Discussion

In this study, we have assessed the COPD’s influence on the severity of clinical and CT characteristics of COVID-19-associated pneumonia. The hospitalization rate of our patients with COPD and COVID-19 infection was relatively low, only 76 out of 281 patients, 50 of which demonstrated CT spectrum of COVID-19-associated pneumonia. This echoes the reports on the relatively low hospitalization rates of the patients with COVID-19 infection in other countries, average 2–12% [1, 11, 26–28]. Also, as per the summary of the Global Initiative for Chronic Obstructive Lung Disease’s 2022, patients with COPD are not at greatly increased risk of infection with SARS-CoV-2 [29].

Our COPD group had a higher mean age and larger male fraction compared to the non-COPD group. This is concordant with demographic data observed by Wu F. et al. and Turan O. et al., with the reported male fraction of 79–83% and mean age of 70–71 years in COPD- vs. 55–56 years for the non-COPD group [30, 31].

The severity of COVID-19 infection and outcomes are reported to be worse in the patients with COPD due to increased expression of angiotensin-converting enzyme-2 receptors (ACE-2 receptors) in the airways and other organs, especially in the presence of diabetes, cardiovascular disease, and obesity [19]. COPD is believed to be an independent factor for respiratory failure in COVID-19 infection given chronic airway dysfunction and increased potential of virus binding [19]. However, hypoxia level, in-hospital mortality, and intensive care unit (ICU) admission rate are heterogeneously reported in the literature. For example, Gemicioglu B. et al. demonstrated a high SpO2 level of 92.57 ± 7.1% in COPD patients with COVID-19 infection [32]. At the same time, Aalinezhad M. et al. has concluded that COPD was linked to more profound hypoxemia in patients with COVID-19-associated pneumonia (89.83 ± 8.02) [33]. Lee et al. in the nationwide retrospective study in South Korea has shown an increased rate of ICU admission, mechanical ventilation and all-cause mortality in the COPD group [34]. In our study, we have found no statistically significant difference in the level of SpO2, hospital length of stay or in-hospital mortality between COPD and non-COPD groups. Our observations are concordant with those by Turan O. et al. who demonstrated a mortality rate of 13.2% vs. 7.% and hospitalization length of 10.06 ± 4.04 days vs. 11.05 ± 5.42 days in COPD- vs. non-COPD group, respectively [31]. Similarly, COPD was not a significant risk factor for increased in-hospital mortality in comparison with non-COPD patients in several other studies [31, 35, 36]. On the contrary, Guan WJ et al. and Lee et al. identified that COPD as a risk factor for ICU admission assisted ventilation and mortality, which remained significant even after the adjustments for age and smoking [16, 34]. Gerayeli FV et al. reported increased odds of hospitalization (OR = 4.23, 95% CI 3.65–4.90), ICU admission (OR = 1.35, 95% CI 1.02–1.78), and mortality (OR = 2.47, 95% CI 2.18–2.79), accompanied by the results of Sanchez-Ramirez DC et al. on higher odds of worse outcomes (OR = 5.8; 95% CI: 3.9–8.5) in patients with COPD [37, 38].

Such varieties in the course of the disease and outcomes between the studies might be multifactorial, and at least partially explained by the unique genetic profile (including expression of ACE-2 receptors), ethnicity and geography, as well as comorbidities, treatment variations, criteria for ICU admission, and mechanical ventilation modes. For instance, a tendency to therapeutic rather than prophylactic anti-coagulation in our country (not published data), even with a milder course of the disease, might have resisted the effects of endothelial cell dysfunction and coagulopathy previously reported in COVID-19 infection [34]. Moreover, severe course of the disease and associated diffuse alveolar damage by CT criteria was rare in our study (2% in COPD- and 2.6% in non-COPD group). Finally, a relatively small number of the included patients and methodology might have impacted our results too.

In regards to the major comorbidities, diabetes mellitus and cardiovascular diseases were observed at the same rate in both groups. This is concordant with other studies [16, 27, 34,]. For example, Marron RM et al. showed increased rates of ischemic heart disease, congestive heart failure and stroke among all the patients with COPD, with no obvious difference in the admission rates in comparison with the patients without COPD [39].

We have observed that dyspnea at rest, dry cough and fever ≥ 38.0** °C** were the most common symptoms on admission in both groups. Guan WJ et al. and Zhang J et al. have demonstrated a similar rate of mild fever (88.0% *vs.*79.5%) and cough (70.2% *vs.* 61.8%) in both groups, respectively [16, 40]. Similarly, Goyal P. et al. concluded that cough (79.4%), fever (77.1%) and dyspnea (56.5%) were the most common clinical findings regardless of the COPD status [28].

Our laboratory values showed lower ALC and higher leukocyte, including ANC and NLR in patients with COPD. Similar results are demonstrated by Gemicioglu B. et al., lower ALC (1.42 ± 0.77 × 109/L), higher WBC (10.6 ± 5.2 × 109/L) and ANC (7.94 ± 4.18 × 109/L), with the median level of NLR 8.09 ± 7.25 [32]. In contrast, Basin S. et al. observed no significant differences in the level of leukocytes and lymphocytes in patients with COPD vs. control group (6.4 × 109/L vs. 6.5 × 109/L and 1.0 vs. 0.9 × 109/L, respectively) [41]. Such heterogeneities in the results might be due to differences in the severity of the disease among included patients in different studies. In our study, only 1 patient from COPD group and 2 patients from non-COPD group had diffuse alveolar damage.

None of our CT findings differ statistically between COPD and non-COPD groups. However, the patients with COPD were more likely to have milder CT severity score, especially CT1 (less than 25% of lung involvement) category, less likely to have bilateral and central localization of GGO, right lower lobe and left lung involvement, as well as a “crazy-paving” pattern. “Reversed halo” sign was not present in the patients with COPD. In regards to the severity score, our results are concordant with those by Basin S. et al. [41]. In detail, the referenced study reported CT1 category of 63.1% vs. 42.4%; CT2 category, 26.2% vs. 40.2%; CT3 category, 9.2% vs. 15.0%; CT4 category (more than 75%), 1.5% vs. 3.3% in COPD vs. non-COPD group, respectively. CT-based severity score of less than 25% of lung involvement (CT1 category) in patients with COPD was the most common in his study, similar to ours. We have found a positive correlation between CT severity score and in-hospital mortality in patients with hypoxia regardless COPD status, with the most significant mortality rate among the patients under CT severity score of 4 (more than 75% of lung involvement). However, we had only 4 patients with CT 4 category in COPD group vs. 30 patients without COPD.

There is a number of limitations in our study. First, spirometry data in the patients with CT findings compatible with COPD are not available. Second, the cohort of the patients with COPD is relatively small, i.e., only 50 patients. Moreover, a small number of the patients included in non-COPD group did report a smoking history but did not have findings of COPD by CT criteria. Third, we did not longitudinal evaluation of the disease severity by CT. Also, inflammatory cytokine levels that might influence the disease severity and outcome were not done at our hospital. However, we did include conventional laboratory values that are known to correlate with the severity of the disease and outcome. Overall, we believe that our results do contribute to the existing literature on clinical and imaging characterization of the patients with COVID-19 infection and suggest that older age, male gender, CT-based severity score, and hypoxia correlate with the clinical severity and in-hospital mortality regardless of COPD status.

## Conclusions

COPD was most often associated with mild severity of COVID-19 infection in our study and did not significantly influence the severity of clinical, conventional laboratory, or CT-based characteristics of COVID-19-associated pneumonia. In addition, the presence of COPD by CT criteria was not linked to the increased in-hospital mortality.

## Data Availability

The datasets used and/or analyzed during the study are available upon reasonable request.

## Abbreviations

SARS-CoV-2: Severe acute respiratory syndrome-related coronavirus 2
COVID-19: Coronavirus disease 2019
COPD: Chronic obstructive pulmonary disease
CT: Computed tomography
RT-PCR: Reverse transcription-polymerase chain reaction
SpO_2_: Arterial oxygen saturation
WBC: White blood cells
ALC: Absolute lymphocyte counts
ANC: Absolute neutrophil count
NLR: Neutrophil/lymphocyte ratio
ALT: Alanine aminotransferase
AST: Aspartate aminotransferase
kVp: Kilovoltage
mAs: Milliampere
mm: Millimeter
HU: Hounsfield unit
GGO: Ground-glass opacities
ORs: Odds ratios
CIs: 95% Confidence intervals
RR: Relative risk
ACE-2 receptors: Angiotensin-converting enzyme-2 receptors
ICU: Intensive care unit
